# Extrapulmonary Tuberculosis by Nationality, the Netherlands, 1993–2001

**DOI:** 10.3201/eid1209.050553

**Published:** 2006-09

**Authors:** Lowieke A.M. te Beek, Marieke J. van der Werf, Clemens Richter, Martien W. Borgdorff

**Affiliations:** * KNCV Tuberculosis Foundation, The Hague, the Netherlands;; † Municipal Health Service Kop van Noord-Holland, Schagen, the Netherlands; .; ‡ Rijnstate Hospital, Arnhem, the Netherlands;; § Academic Medical Centre, University of Amsterdam, Amsterdam, the Netherlands

**Keywords:** tuberculosis, nationality, epidemiology, the Netherlands, pleural tuberculosis, lymph node tuberculosis

## Abstract

This study describes the epidemiology of extrapulmonary tuberculosis (TB) in the Netherlands from 1993 through 2001. We assessed whether the increasing numbers of inhabitants with a non-Western ethnic background had an effect on the number of extrapulmonary patients. We used data from the Netherlands Tuberculosis Register and included all cases of TB diagnosed in the Netherlands between January 1, 1993, and December 31, 2001. Information on age, sex, nationality, year of diagnosis, culture result, anatomic location of the site of disease, and HIV status was retrieved from the register. Of 13,258 patients with TB, 8,216 (62%) had pulmonary TB, and 5,042 (38%) had extrapulmonary TB. Non-Dutch nationals were more likely to have most types of extrapulmonary TB. The growth of the number of inhabitants with a non-Western ethnic background in the Netherlands explains the proportional growth of extrapulmonary TB. Physicians need to be aware of the changing clinical picture of TB.

## Abstract 2

The growth of the number of inhabitants with a non-Western ethnic background most likely explains the growth of extrapulmonary TB in the Netherlands.

Tuberculosis (TB) is a major public health problem, affecting 8 million persons per year worldwide (*1*). The global incidence rate of TB per capita is growing by ≈1.1% per year (*1*). Contrary to the increasing number of TB cases in developing countries, the number of cases in industrialized countries is stable or decreasing (*2**–**4*). In the United States, a decreasing trend of the total number of TB patients is seen with an increasing proportion of TB cases with extrapulmonary TB, resulting in a rising proportion from 7.8% in 1964 to 20% in 2001 (*5**–**8*). Both the HIV epidemic and changes in population demographics, with rising numbers of immigrants, are being held responsible for this proportional increase of extrapulmonary TB (*6**,**7**,**9*). A recent study of extrapulmonary TB in Hong Kong Special Administrative Region, People's Republic of China showed that 22.3% of the TB cases were extrapulmonary (*10*), while a small Canadian study found a proportion of 46% (*11*). No national studies about extrapulmonary TB in developing countries are known.

Extrapulmonary TB refers to TB outside the lungs. Mycobacteria may spread through lymphatic or hematogenous dissemination to any tract or through coughing and swallowing to the gastrointestinal tract. Bacteria may remain dormant for years at a particular site before causing disease. Since extrapulmonary TB can affect virtually all organs, it has a wide variety of clinical manifestations, which causes difficulty and delay in diagnosis (*7**,**8*). Obtaining material for culture confirmation of extrapulmonary TB is much more difficult than obtaining material for culture confirmation of pulmonary TB (*7*). Extrapulmonary TB is more often diagnosed in women and young patients (*6**,**7**,**9**–**13*). In the United States, extrapulmonary TB is associated with ethnic minorities and those born in other countries (*6*). In many countries, patients from Asian origin are known to have a higher incidence of extrapulmonary TB, especially lymphatic TB (*11**,**14**–**17*). A study of Somali TB patients in Minnesota showed frequent lymphatic TB as well (*18*). In HIV-infected patients the frequency of extrapulmonary TB depends on the degree of decreased cellular immunity (*19**,**20*). In patients with <100 CD4 cells/mL, extrapulmonary and disseminated TB counts for 70% of all forms of TB (*21*).

The Netherlands has a low incidence of HIV and, similar to other industrialized countries, a decreasing TB incidence rate. TB was a common disease in the nineteenth century, but after the Second World War the incidence rate declined rapidly (*22*). During the last decade, the numbers of inhabitants with a non-Western ethnic background has been growing (*23*). The proportion of TB patients with a non-Dutch nationality has increased from 30% in 1980 to 63% in 2000 (*22**,**24**,**25*). Dutch policy regulates the screening by chest radiograph of immigrants from countries with a high prevalence who intend to stay for more than 3 months (*26*). No studies have described the epidemiology of extrapulmonary TB in the Netherlands, with the exception of 1 study on bone and joint TB (*27*).

This article describes the epidemiology of extrapulmonary TB in the Netherlands between 1993 and 2001. Our main focus is on the relation between extrapulmonary TB and nationality. We assess whether the increasing number of inhabitants with a non-Western ethnic background has had an effect on the number of extrapulmonary patients.

## Methods

For this study, we used data from the Netherlands Tuberculosis Register (NTR), an anonymous case register held by KNCV Tuberculosis Foundation. Every TB patient reported to 1 of the 45 municipal health services in the Netherlands is included in this register. Reporting to the register is voluntary. Comparison with the mandatory notification system of the Ministry of Health suggests 99% completeness (*28*). We included all cases of TB diagnosed in the Netherlands from January 1, 1993 through December 31, 2001. Information on age, sex, and nationality, as recorded in the passport, year of diagnosis, culture result, anatomic location of the site of disease, and HIV status was retrieved from the register. Non-Dutch nationality was divided into 7 different nationality groups: Europe (central and eastern: Poland, Czech, Slovakia, Hungary, Romania, Bulgaria, Albania and the countries of the former Yugoslavia and the former Soviet Union), Turkey, Morocco, Somalia, Africa (other), Asia, and other countries. HIV testing is not a standard procedure for TB patients in the Netherlands. We considered case-patients with a record of impaired immunity due to HIV infection as HIV infected and all others as HIV status unknown.

Information about the anatomic location of disease was registered using 2 different classification systems. In the NTR, the location of TB is generally divided into 3 categories, pulmonary, extrapulmonary, or combined pulmonary and extrapulmonary disease, and the disease is classified according to the site of the disease by using the International Classification of Diseases 9th revision (ICD-9). We first used the information from the ICD-9 classification to divide the patients into a pulmonary TB group, i.e., major site of disease location inside the lungs, and an extrapulmonary TB group, i.e., the major site of disease was outside the lungs. Thereafter, 8 types of extrapulmonary disease were defined: lymphatic, pleural, bone and/or joint, genitourinary, miliary, peritoneal, meninges and/or central nervous system (CNS), and all other sites combined. If information from the ICD-9 classification was not available, we used the general information about location of the disease. Case-patients with general location pulmonary disease were allocated to the pulmonary TB group and those with extrapulmonary disease were allocated to the extrapulmonary TB group, site of disease unspecified. Patients for whom no information about the ICD-9 classification was available, and with the general location of combined pulmonary and extrapulmonary disease, were excluded from the analysis.

Each patient was included once in our dataset. If a patient was reported to the register with TB more than once from January 1, 1993, through December 31, 2001, only the information from the first registration was included in the dataset. Patients with a culture diagnosis of *Mycobacterium bovis* and *M. bovis* BCG were excluded as were patients with missing data for age, sex, or nationality.

We obtained the total number of inhabitants with Dutch and non-Dutch nationality in the Netherlands from January 1, 1993, through January 1, 2002, from the Central Bureau of Statistics of the Netherlands (*29*). We used these figures as denominators to assess trends in time of the incidence of extrapulmonary and pulmonary TB by Dutch or non-Dutch nationality with Poisson regression. One-way analysis of variance (ANOVA) was used to analyze differences in means of age. We used the χ^2^ test to compare the proportions of pulmonary and extrapulmonary TB cases and non parametric tests to compare time to TB diagnosis from immigration for foreign born persons. Differences between pulmonary TB and the 8 separate types of extrapulmonary TB, were tested by using logistic regression, with pulmonary disease as the reference category. The variables age, sex, nationality and HIV status were included in a multivariate logistic regression model to adjust for confounding. Statistical analysis was performed by using SPSS version 11.5 for Windows (SPSS Inc, Chicago, IL, USA).

## Results

Between January 1993 and December 2001, 13,943 cases of TB were reported to the Netherlands Tuberculosis Register, 10,688 (76.7%) were culture positive. The 270 case-patients that were reregistrations were excluded. Also excluded were 135 patients who had a diagnosis of *M. bovis* or *M. bovis* BCG infection; 190 patients with missing information about age, sex, or nationality; and 90 patients with a missing ICD-9 classification and combined pulmonary and extrapulmonary disease.

Of the total of 13,258 (95.1%) TB patients available for analysis, 8,216 (62.0%) had pulmonary TB, and 5,042 (38.0%) had an extrapulmonary major site of disease (Table 1). The proportion with culture confirmation was slightly larger among the pulmonary (71.5%) than among the extrapulmonary TB patients (67.4%) (χ^2^ test, p<0.01). The male-to-female ratio was 1.5:1. Male TB patients were slightly younger than female TB patients (ANOVA, p<0.01), the mean age for men was 39.0 years and for women, 40.2 years. Most TB patients (57.1%) were non-Dutch. Of the 7,576 non-Dutch patients, 1,692 (22.3%) were Somali, 1,600 (21.1%) were Asian, 1,267 (16.7%) were Moroccan, and 1,245 (16.4%) were African (other). Asians included 32 different nationalities; the largest number of patients was from Pakistan (243 cases), followed by Indonesia (239 patients) and the People's Republic of China (179 patients). The African (other) group consisted of 48 nationalities with the largest numbers of patients being from Ethiopia (163 patients) and Angola (146 patients). Patients from Somalia, Asia, and Morocco more frequently had a diagnosis of extrapulmonary TB then did Dutch patients, whereas patients from central and eastern Europe had extrapulmonary TB less often (χ^2^ test, p<0.01). Non-Dutch patients were significantly younger than Dutch patients (ANOVA p<0.01), the mean ages were 31.7 and 49.8 years, respectively. The male-to-female ratio was 1.4:1 among Dutch patients and 1.5:1 among non-Dutch patients (χ^2^ test, p<0.01). Among the non-Dutch, 41.7% of the TB patients had extrapulmonary TB, compared with 33.1% of the Dutch TB patients (p<0.01). No interaction was found between the variables age, nationality, sex, and HIV status.

**Table 1:** General characteristics of pulmonary and extrapulmonary tuberculosis (TB) patients in the Netherlands, 1993–2001

		Extrapulmonary TB (%)	Pulmonary TB (%)	Total (%)	p value*
Total		5,042 (38.0)	8,216 (62.0)	13,258 (100)	
Sex					<0.01
	Male	2,610 (33.1)	5,285 (66.9)	7,895 (100)	
	Female	2,432 (45.3)	2,931 (54.7)	5,363 (100)	
Age groups, y					<0.01
	<14	255 (31.4)	556 (68.6)	811 (100)	
	15–24	877 (36.1)	1,553 (63.9)	2,430 (100)	
	25–34	1,389 (39.3)	2,149 (60.7)	3,538 (100)	
	35–44	810 (38.0)	1,321 (62.0)	2,131 (100)	
	45–64	893 (40.9)	1,293 (59.1)	2,186 (100)	
	>65	818 (37.8)	1,344 (62.2)	2,162 (100)	
HIV infection†					<0.01
	Yes	173 (34.7)	325 (65.3)	498 (100)	
	Unknown	4,869 (38.2)	7,891 (61.8)	12,760 (100)	
Nationality					<0.01
	Dutch	1,882 (33.1)	3,800 (66.9)	5,682 (100)	
	European, central and eastern	70 (15.1)	395 (84.9)	465 (100)	
	Turkish	212 (32.1)	449 (67.9)	661 (100)	
	Moroccan	509 (40.2)	758 (59.8)	1,267 (100)	
	Somali	997 (58.9)	695 (41.1)	1,692 (100)	
	African (other)	456 (36.6)	789 (63.4)	1,245 (100)	
	Asian	705 (44.1)	895 (55.9)	1,600 (100)	
	Other	211 (32.7)	435 (67.3)	646 (100)	
Culture determination					<0.01
	Positive	3,400 (36.7)	5,872 (63.3)	9,275 (100)	
	Negative	188 (51.8)	175 (48.2)	363 (100)	
	Unknown or not performed	1,454 (40.2)	2,166 (59.8)	3,620 (100)	

*p value (2-sided) for proportions by Pearson χ^2^ †HIV testing is not a standard procedure for TB patients in the Netherlands. HIV infection is recorded in the National Tuberculosis Register as one of the response options of the item "impaired immunity." Considered as HIV infected were those cases with a record of impaired immunity due to HIV infection; all others were classified as HIV status unknown.

Between 1993 and 2001 the total number of pulmonary TB cases decreased (rate ratio per year 0.96, 95% confidence interval [CI] 0.95–0.96; p<0.01), especially among the Dutch population (Figure). The total number of extrapulmonary patients showed no significant change in time (rate ratio per year 1.00, 95% CI 0.99–1.02; p = 0.40), but the number of extrapulmonary TB patients with a Dutch nationality decreased (rate ratio per year 0.96, 95% CI 0.94–0.98; p<0.01), while the number of extrapulmonary TB patients with a non-Dutch nationality increased (rate ratio per year 1.06, 95% CI 1.04–1.07; p<0.01).

**Figure:**
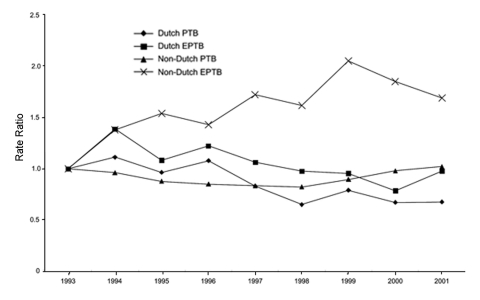
Relative incidence of pulmonary tuberculosis (PTB) and extrapulmonary tuberculosis (EPTB) among Dutch and non-Dutch residents, the Netherlands, 1993–2001.

Extrapulmonary TB was relatively more prevalent among female (45.3%) than among male TB patients (33.1%) (p<0.01). The mean age of extrapulmonary TB patients was slightly higher (40.3 years) than that of pulmonary cases (39.0 years; ANOVA p<0.01). The most frequent types of extrapulmonary TB were lymphatic TB (1,963 cases), pleural TB (1,036 cases), and TB of the bones and/or joints (465 cases) (Table 2). Other sites accounted for 111 (TB of meninges and/or CNS) to 246 (miliary TB) cases, while for 379 of the extrapulmonary cases, site of disease was not recorded. The proportion of HIV-infected patients ranged from 0.9% (genitourinary TB) to 16.7% (miliary TB), depending on the site of disease (Table 3). The median time since immigration into the Netherlands to diagnosis of TB among non-Dutch was 36.0 months (mean 73.0 months) and varied from 7.9 months in central and eastern Europeans to 9.0 years in Moroccans. Among all non-Dutch pulmonary TB had a shorter time from immigration to diagnosis (median 24.0 months) than extrapulmonary TB (median 44.6 months) (Mann-Whitney U test p<0.01).

**Table 2:** Distribution of total tuberculosis (TB) and EPTB by nationality and culture determination in the Netherlands, 1993–2001*†

		Total TB‡ (%)	Total EPTB (%)	Lymphatic (%)	Pleural (%)	Bones and joints (%)	Miliary (%)	Genitourinary (%)	Peritoneal (%)	Meninges and CNS (%)	Other EPTB (%)
Total		13,258 (100)	5,042 (38.0)	1,963 (14.8)	1,036 (7.8)	465 (3.5)	246 (1.9)	226 (1.7)	178 (1.3)	111 (0.8)	438 (3.3)
Nationality											
	Dutch	5,682 (100)	1,882 (33.1)	441 (3.3)	592 (4.5)	188 (1.4)	136 (1.0)	147 (1.1)	34 (0.3)	52 (0.4)	133 (1.0)
	European (central and eastern)	465 (100)	70 (15.1)	23 (4.9)	26 (5.6)	1 (0.2)	7 (1.5)	3 (0.6)	1 (0.2)	0	4 (0.9)
	Turkish	661 (100)	212 (32.1)	88 (13.3)	45 (6.8)	7 (1.1)	4 (0.6)	12 (1.8)	7 (1.1)	3 (0.5)	24 (3.6)
	Moroccan	1,267 (100)	509 (40.2)	233 (18.4)	87 (6.9)	29 (2.3)	14 (1.1)	24 (1.9)	29 (2.3)	12 (0.9)	46 (3.6)
	Somali	1,692 (100)	997 (58.9)	497 (29.4)	84 (5.0)	107 (6.3)	22 (1.3)	11 (0.7)	58 (3.4)	20 (1.2)	110 (6.5)
	African (other)	1,245 (100)	456 (36.6)	189 (15.2)	83 (6.7)	43 (3.5)	32 (2.6)	12 (1.0)	12 (1.0)	5 (0.4)	49 (3.9)
	Asian	1,600 (100)	705 (44.1)	407 (25.4)	75 (4.7)	64 (4.0)	19 (1.2)	9 (0.6)	27 (1.7)	16 (1.0)	57 (3.6)
	Other	646 (100)	211 (32.7)	85 (13.2)	44 (6.8)	26 (4.0)	12 (1.9)	8 (1.2)	10 (1.5)	3 (0.5)	15 (2.3)
Culture determination											
	Positive	9,275 (100)	3,400 (36.7)	1,402 (15.1)	683 (7.4)	362 (3.9)	185 (2.0)	167 (1.8)	141 (1.5)	74 (0.8)	288 (3.1)
	Negative	363 (100)	188 (51.8)	80 (22.0)	59 (16.3)	13 (3.6)	2 (0.6)	5 (1.4)	3 (0.8)	6 (1.7)	16 (4.4)
	Unknown or not performed	3,620 (100)	1,454 (40.2)	481 (13.3)	294 (8.1)	90 (2.5)	59 (1.6)	54 (1.5)	34 (0.9)	31 (0.9)	134 (3.7)

*EPTB, extrapulmonary TB; CNS, central nervous system. †The denominator for percentages is the total TB cases in the row. ‡Total TB includes both PTB and EPTB.

**Table 3:** Distribution of total tuberculosis (TB) and EPTB by sex, age group, and HIV diagnosis category in the Netherlands, 1993–2001*†

		Total TB‡ (%)	Total EPTB (%)	Lymphatic (%)	Pleural (%)	Bones and joints (%)	Miliary (%)	Genitourinary (%)	Peritoneal (%)	Meninges and CNS (%)	Other EPTB (%)
Total		13,258 (100)	5,042 (38.0)	1,963 (14.8)	1,036 (7.8)	465 (3.5)	246 (1.9)	226 (1.7)	178 (1.3)	111 (0.8)	438 (3.3)
Sex											
	Male	7,895 (100)	2,610 (33.1)	904 (11.5)	676 (8.6)	224 (2.8)	136 (1.7)	121 (1.5)	78 (1.0)	69 (0.9)	219 (2.8)
	Female	5,363 (100)	2,432 (45.3)	1,059 (19.7)	360 (6.7)	241 (4.5)	110 (2.1)	105 (2.0)	100 (1.9)	42 (0.8)	219 (4.1)
Age group, y											
	<14	811 (100)	255 (31.4)	133 (16.4)	32 (3.9)	14 (1.7)	6 (0.7)	0	3 (0.4)	13 (1.6)	23 (2.8)
	15–24	2,430 (100)	877 (36.1)	373 (15.3)	204 (8.4)	66 (2.7)	26 (1.1)	10 (0.4)	41 (1.7)	10 (0.4)	82 (3.4)
	25–34	3,538 (100)	1,389 (39.3)	608 (17.2)	312 (8.8)	105 (3.0)	40 (1.1)	30 (0.8)	50 (1.4)	31 (0.9)	121 (3.4)
	35–44	2,131 (100)	810 (38.0)	347 (16.3)	140 (6.6)	75 (3.5)	34 (1.6)	37 (1.7)	37 (1.7)	13 (0.6)	77 (3.6)
	45–64	2,186 (100)	893 (40.9)	301 (13.8)	165 (7.5)	97 (4.4)	45 (2.1)	75 (3.4)	34 (1.6)	28 (1.3)	77 (3.5)
	>65	2,162 (100)	818 (37.8)	201 (9.3)	183 (8.5)	108 (5.0)	95 (4.4)	74 (3.4)	13 (0.6)	16 (0.7)	58 (2.7)
HIV infection§											
	Yes	498 (100)	173 (34.7)	60 (12.0)	18 (3.6)	9 (1.8)	41 (8.2)	2 (0.4)	8 (1.6)	10 (2.0)	21 (4.2)
	Unknown	12,760 (100)	4,869 (100)	1,903 (14.9)	1,018 (8.0)	456 (3.6)	205 (1.6)	224 (1.8)	170 (1.3)	101 (0.8)	417 (3.3)

*EPTB, extrapulmonary TB; CNS, central nervous system. †The denominator for percentages is the total TB cases in the row. ‡Total TB includes both PTB and EPTB. §HIV testing is not a standard procedure for TB patients in the Netherlands. HIV infection is recorded in the National Tuberculosis Register as one of the response options of the item "impaired immunity." Considered as HIV infected were those cases with a record of impaired immunity due to HIV infection; all others were classified as HIV status unknown.

The genitourinary tract was the site of TB that had the smallest proportion of non-Dutch patients (34.2%) while 80.9% of the patients with peritoneal TB were non-Dutch. Somali TB patients had a strongly increased frequency of peritoneal TB (crude odds ratio 9.4, adjusted odds ratio [AOR] 12, 95% CI 7.6–20), lymphatic TB (Crude odds ratio 7.2, AOR 7.8, 95% CI 6.6–9.3) and TB of bones and/or joints (crude odds ratio 3.5, AOR 6.1, 95% CI 4.5–8.3) than the Dutch (Table 4). Asian TB patients had a higher frequency of lymphatic TB (crude odds ratio 4.1, AOR 4.2, 95% CI 3.6–5.0) and peritoneal TB (crude odds ratio 3.2, AOR 3.4, 95% CI 2.0–5.9) than the Dutch. The proportion of TB of the bones and/or joints, genitourinary TB, and miliary TB compared to pulmonary TB increased with the age of the patients. Both the age groups <14 years and 45–64 years were associated with TB of the meninges and/or CNS, in univariate analysis (crude odds ratio 3.9 and 1.8, respectively). When the study population was stratified in <14 and >15 years age groups, all types of extrapulmonary TB were associated with the oldest age group, except for TB of the meninges and/or CNS, which was weakly associated with the youngest age group (AOR 1.7, 95% CI 0.9–3.1). HIV infection was positively associated with miliary TB (AOR 7.4, 95% CI 4.9–11) and TB of the meninges and/or CNS (AOR 3.3, 95% CI 1.6–6.7) and negatively associated with pleural and genitourinary TB.

**Table 4:** Risk factors for 8 types of extrapulmonary tuberculosis (TB) versus pulmonary TB*†

		Lymphatic (n = 1,963)	Pleural (n = 1,036)	Bones and joints (n = 465)	Miliary (n = 246)	Genitourinary (n = 226)	Peritoneal (n =178)	Meninges and CNS (n = 111)	Other EPTB sites (n = 438)
Sex									
	Male	1.00	1.00	1.00	1.00	1.00	1.00	1.00	1.00
	Female	**2.22 (2.00–2.46)**	0.94 (0.82–1.08)	**1.95 (1.61–2.37)**	**1.56 (1.20–2.03)**	**1.70 (1.29–2.22)**	**2.55 (1.87–3.46)**	1.08 (0.73–1.60)	**1.93 (1.59–2.36)**
Age group, y									
	<14	**0.68 (0.52–0.89)**	**0.52 (0.35–0.77)**	**0.16 (0.09–0.29)**	**0.10 (0.04–0.23)**	–	**0.18 (0.05–0.66)**	1.54(0.70–3.36)	**0.44 (0.26–0.75)**
	15–24	**0.63 (0.51–0.78)**	**1.34 (1.06–1.68)**	**0.27 (0.18–0.38)**	**0.14 (0.08–0.23)**	**0.12 (0.06–0.25)**	0.90 (0.45–1.81)	0.43 (0.18–1.01)	**0.54 (0.37–0.81)**
	25–34	0.86 (0.70–1.05)	**1.51 (1.22–1.86)**	**0.36 (0.26–0.50)**	**0.14 (0.09–0.22)**	**0.29 (0.18–0.47)**	0.94 (0.48–1.85)	0.97 (0.49–1.90)	**0.68 (0.47–0.98)**
	35– 44	1.03 (0.83–1.27)	1.05 (0.82–1.34)	**0.56 (0.40–0.78)**	**0.20 (0.13–0.33)**	**0.62 (0.40–0.95)**	1.58 (0.80–3.13)	0.70 (0.32–1.53)	0.92 (0.63–1.35)
	45–64	1.11 (0.90–1.37)	1.08 (0.86–1.35)	0.85 (0.63–1.15)	**0.41 (0.28–0.60)**	1.16 (0.83–1.64)	1.85 (0.95–3.62)	1.71 (0.91–3.23)	1.13 (0.78–1.63)
	>65	1.00	1.00	1.00	1.00	1.00	1.00	1.00	1.00
HIV infection‡									
	Yes	0.92 (0.68–1.24)	**0.40 (0.25–0.65)**	0.52 (0.26–1.03)	**7.36 (4.88–11.12)**	**0.25 (0.06–1.04)**	1.41 (0.66–3.01)	**3.29 (1.63–6.65)**	1.36 (0.85–2.19)
	Unknown	1.00	1.00	1.00	1.00	1.00	1.00	1.00	1.00
Nationality									
	Dutch	1.00	1.00	1.00	1.00	1.00	1.00	1.00	1.00
	European (central and eastern)	**0.57 (0.37–0.88)**	**0.37 (0.25–0.56)**	**0.08 (0.01–0.54)**	1.06 (0.48–2.33)	0.31 (0.10–0.98)	0.31 (0.04–2.32)	–	**0.35 (0.13–0.96)**
	Turkish	**2.01 (1.55–2.61)**	**0.55 (0.40–0.76)**	0.53 (0.25–1.15)	0.64 (0.23–1.77)	1.27 (0.68–2.38)	2.19 (0.95–5.09)	0.63 (0.19–2.07)	**2.01 (1.26–3.19)**
	Moroccan	**2.98 (2.47–3.60)**	**0.67 (0.53–0.86)**	1.11 (0.73–1.68)	1.12 (0.63–2.00)	1.23 (0.78–1.96)	**4.71 (2.78–7.97)**	1.31 (0.68–2.52)	**2.08 (1.45–2.99)**
	Somali	**7.82 (6.56–9.31)**	**0.68 (0.53–0.88)**	**6.10 (4.51–8.26)**	**2.75 (1.61–4.67)**	1.14 (0.59–2.22)	**12.47 (7.64–20.36)**	**2.82 (1.57–5.07)**	**6.39 (4.69–8.70)**
	African (other)	**2.79 (2.27–3.42)**	**0.61 (0.47–0.79)**	**2.32 (1.59–3.39)**	**2.28 (1.41–3.66)**	1.09 (0.58–2.07)	**2.21 (1.09–4.46)**	0.53 (0.20–1.39)	**2.48 (1.71–3.60)**
	Asian	**4.19 (3.55–4.96)**	**0.47 (0.36–0.61)**	**2.06 (1.50–2.83)**	1.28 (0.77–2.15)	**0.42 (0.21–0.84)**	**3.44 (2.01–5.91)**	1.63 (0.89–2.95)	**2.09 (1.49–2.94)**
	Other	**1.78 (1.37–2.31)**	**0.63 (0.46–0.88)**	**1.70 (1.08–2.59)**	1.03 (0.55–1.94)	0.67 (0.32–1.39)	**2.44 (1.17–5.07)**	0.47 (0.14–1.54)	1.05 (0.60–1.83)

*EPTB, extrapulmonary tuberculosis; PTB, pulmonary TB. †In the calculation of odds ratios for patients, patients with PTB served as a reference group. The odds ratios were adjusted for all variables used in the model. **Boldface** figures denote significant odds ratios. ‡HIV testing is not a standard procedure for TB patients in the Netherlands. HIV infection is recorded in the National Tuberculosis Registry as one of the response options of the item "impaired immunity." Considered as HIV infected were those cases with a record of impaired immunity due to HIV infection; all others were classified as HIV-status unknown.

## Discussion

In the Netherlands, the number of inhabitants with a non-Western ethnic background increased over the past 2 decades (*23*). The Netherlands is likely to remain an immigration destination for persons from non-Western countries, although changes in immigration laws can change this situation (*23**,**24*). We assessed the effect of these immigration patterns on the incidence of pulmonary and extrapulmonary TB.

Between 1993 and 2001, the number of pulmonary TB cases per year declined, whereas the number of extrapulmonary TB cases remained stable, thus showing a proportional increase. This trend is similar to the trend observed in studies in the United States (*6**,**7*). Our study shows that a non-Dutch nationality, especially Somali and Asian, was positively associated with extrapulmonary TB when compared with the results for pulmonary TB. This finding suggests that the most likely explanation for the proportional increase of extrapulmonary TB is the growth of the number of inhabitants with a non-Western ethnic background.

Our analysis showed that persons from non-Western national groups, especially Somalis, Asians, and Moroccans, were more likely to receive a diagnosis of most types of extrapulmonary TB than Dutch nationals. When looking at the absolute number of patients with lymphatic and peritoneal TB exclusively, patients with Somali nationality even outnumbered Dutch patients (497 and 58 vs 441 and 34, respectively). In agreement with the literature, we found a strongly positive association between Somali nationality and lymphatic and bone and/or joint TB (*18**,**27*) and between Asian nationalities and lymphatic TB (*6**,**10**,**11**,**16*). Our study demonstrated a statistically significant, strong, positive association between peritoneal TB and Somali, Moroccan, or Asian nationality. In contrast, pleural TB was significant negatively associated with the non-Dutch patients when results were compared with those of the Dutch patients, a finding that we cannot explain.

Several explanations are possible for the association of extrapulmonary TB and a non-Dutch nationality. Non-Dutch persons may have a higher frequency of extrapulmonary TB due to an impaired immunity caused by factors such as vitamin D deficiency (*11**,**16**,**30**,**31*), dietary changes (*16*), and restricted social conditions (*16**,**18*), which cause an endogenous TB infection to reactivate from extrapulmonary or pulmonary sites. Also genetic factors (*32*), for example the presence of polymorphism of the *NRAMP1* gene (*33**,**34*) may contribute to differences in the susceptibility to acquire extrapulmonary TB. Furthermore, *M. tuberculosis* strains circulating outside the Netherlands may be genetically different from those circulating in the Netherlands and cause more extrapulmonary TB.

A limitation in our study is the use of different methods of case finding, since immigrants from countries with a high prevalence are screened for pulmonary TB, but not for extrapulmonary TB (*26*). This circumstance could lead to a possible underestimation of extrapulmonary TB among immigrants, which would make the relationships found in this study even stronger. On the other hand, selection bias is possible when physicians disproportionately suspect and diagnose extrapulmonary TB among certain groups of patients such as non-Western patients. By including 97.0% of all reported new cases of TB in the analysis (13,258 of 13,673 new cases), we tried to minimize bias on inclusion in the study. Fewer patients with extrapulmonary TB had a positive culture than did patients with pulmonary TB (67.4% vs 71.5%). Thus patients with extrapulmonary TB may be misdiagnosed. However, a sensitivity analysis, which included only the culture-positive cases, did not change the conclusions of the logistic regression analysis. Also misdiagnosis of *M. bovis* as *M. tuberculosis* may have occurred in cases in which no culture result was available. Of the 114 *M. bovis* patients excluded from the analysis, all locations of TB were identified: 7 patients had pleural TB, 30 had lymphatic TB, 2 had meningeal TB, 3 had peritoneal TB, 8 had TB of bones and/or joints, 8 had genitourinary TB, 5 had miliary TB, 5 had other extrapulmonary TB, 42 had pulmonary TB, and 4 had extrapulmonary TB not specified.

It should be noted that nationality in the NTR is not always recorded based on passport; sometimes ethnic background is used. Furthermore, a difference in the definition of extrapulmonary TB used in different studies complicates mutual comparison.

Many studies suggest that HIV-induced immunosuppression is associated with extrapulmonary disease (*7**,**35**–**39*), especially with lymphatic and miliary TB (*38**,**39*). In the Netherlands, HIV testing is not a standard procedure for TB patients, which may have led to a possible selection bias in our study. A possible explanation for the absence of associations between HIV infection and most types of extrapulmonary TB in our study can be found in the introduction of the highly active antiretroviral therapy (HAART) in 1996 (*40*). HIV-infected patients treated with HAART will have less impaired immunity (*39*).

Diagnosing extrapulmonary TB can be difficult, so a high index of suspicion remains important. In immigrants from countries with highly endemic TB, a medical history, physical examination, basic laboratory tests, and chest radiograph can lead to a diagnosis. Since TB can occur in all organs, further examination depends on the possible site of infection.

In summary, our analysis showed that there is a proportional increase of extrapulmonary TB in the Netherlands. The growth of the number of inhabitants with a non-Western ethnic background in the Netherlands explains the proportional growth of extrapulmonary TB. Increased awareness among physicians about the changing clinical picture and up-to date knowledge about diagnosis of TB is warranted.
